# Chronic rhinosinusitis association with small airway disease: Inflammatory biomarkers and lung function using impulse oscillometry

**DOI:** 10.1016/j.bjorl.2026.101842

**Published:** 2026-06-16

**Authors:** Mariana Dalbo Contrera Toro, Carla Cristina Souza Gomez, Paloma Lopes Francisco Parazzi, Maria Ângela Gonçalves Oliveira Ribeiro, André Moreno Morcillo, José Dirceu Ribeiro, Eulália Sakano

**Affiliations:** a Universidade Estadual de Campinas, Faculdade de Ciências Médicas, Department of Otolaryngology, Campinas, SP, Brazil; b Universidade Estadual de Campinas, Faculdade de Ciências Médicas, Department of Pediatrics, Pediatrics Investigation Center, Pulmonary Physiology Laboratory, Campinas, SP, Brazil; c Universidade Estadual de Campinas, Faculdade de Ciências Médicas, Department of Pediatrics, Campinas, SP, Brazil

**Keywords:** Rhinosinusitis, Respiratory Function Tests, Nasal polyps, Diagnostic techniques, Respiratory system

## Abstract

**Objective:**

This study aimed to evaluate the correlation between CRS-related biomarkers and disease severity with spirometric and impulse oscillometry parameters in patients with and without asthma.

**Methods:**

We conducted a cross-sectional study involving 52 adult patients with CRS with nasal polyps. All participants underwent clinical evaluation, SNOT-22 scoring, nasal endoscopy, Lund-Kennedy (LK) and Lund-Mackay (LM) scores, blood sampling for eosinophils and IgE, skin prick testing, spirometry, and impulse oscillometry testing. Correlations among upper airway severity, inflammatory markers, and lung function parameters were analyzed using Spearman's rank correlation coefficient.

**Results:**

Patients had preserved mean FVC (Forced Vital Capacity) and FEV1 (Forced Expiratory Volume in 1 s) values, as well as FEV1/FVC (mean 73.6%) and FEF25–75%. Impulse oscillometry showed increased Resonant Frequency and area of reactance. SNOT-22 correlated negatively with FEV1 and FVC, and positively with Z5 (total impedance). Eosinophils correlated negatively with FEV1, FEV1/FVC, and FEF25–75%, and positively with AX (reactance area) and peripheral resistance. No significant correlations were found with total IgE or aeroallergen sensitization.

**Conclusions:**

Impulse oscillometry parameters were similar to those reported in the literature for small airway disease. This study demonstrated a negative correlation between spirometry parameters and blood eosinophil count. There was also a negative correlation between the severity of CRS symptoms and FEV1 and FVC.

**Level of evidence:**

Oxford Centre for Evidence-Based Medicine 2011. Levels of Evidence: Level 4.

## Highlights

• Blood eosinophils correlated negatively with FEV1, FEV1/FVC ratio, and FEF25–75%.
• Impulse oscillometry parameters were compatible with small airway disease in CRS patients.
• A higher SNOT-22 score was associated with lower FVC, FEV1, and Z5.
• Total IgE and aeroallergen sensitization showed no correlation with spirometry or impulse oscillometry.

## Introduction

The unified airway theory helps doctors understand the common pathophysiology of inflammatory upper and lower airway diseases.^1^ Chronic rhinosinusitis, especially type 2 eosinophilic disease, is highly associated with asthma.^2^^,^^3^ However, despite the shared immunological mechanisms, the manifestations of upper and lower airways vary, and different criteria are used to phenotype these diseases.^3^, ^4^, ^5^ However, if those patients can be treated with systemic drugs, such as steroids or immunobiologics, a broad review of the unified airways can be helpful.^1^^,^^6^^,^^7^ There is a need to learn the unique attributes in the upper and lower airway that can lead to distinct clinical and biomarker characteristics in patients with chronic respiratory inflammatory diseases.^8^,^9^

Despite the unified airway hypothesis, there is a lack of studies assessing pulmonary function in patients with Chronic Rhinosinusitis (CRS), particularly in those without a concomitant asthma diagnosis. Previous studies focus predominantly on patients undergoing Functional Endoscopic Sinus Surgery (FESS), emphasizing postoperative spirometric improvement but failing to thoroughly explore preoperative functional variability or its correlation with inflammatory biomarkers.^10^^,^^11^ Recent evidence suggests subclinical impairments in pulmonary function, even in the absence of overt respiratory symptoms, particularly in eosinophilic Chronic Rhinosinusitis (CRS), indicating possible involvement of the distal airways.^10^, ^11^, ^12^ This gap highlights the need for research that integrates detailed pulmonary function assessment into the phenotypic characterization of CRS patients, contributing to a truly integrated approach to airway disease.^11^^,^^13^^,^^14^ Impulse oscillometry is a non-invasive technique that evaluates airway resistance and reactance using pressure oscillations during tidal breathing.^15^^,^^16^ It is particularly effective in detecting subtle alterations in the small airways that may not be evident on conventional spirometry, and thus was chosen to complement the lung function assessment in this study.^17^^,^^18^

This study aimed to investigate whether the inflammatory biomarkers most commonly used to evaluate chronic rhinosinusitis, when used to assess disease severity, are correlated with lower airway obstruction.

## Methods

An observational cross-sectional study was performed between January 2019 and December 2022. The State University of Campinas- UNICAMP Ethical Committee (CAAE: 90802318.3.0000.5404) approved this study, and all patients signed the informed consent.

In this study, adult patients diagnosed with chronic rhinosinusitis with nasal polyps, the primary diffuse kind according to the EPOS classification,^3^ who were followed in the otorhinolaryngology outpatient clinic, were invited to participate. Patients with cystic fibrosis, primary ciliary dyskinesia, or who could not perform spirometry were excluded. Patients with current acute sinusitis or asthma exacerbation symptoms were rescheduled.

All patients answered a clinical questionnaire that included: basic demographic characteristics, the time of diagnosis of CRS, a history of pulmonary and non-pulmonary comorbidities, and intolerance to aspirin, dipyrone, or nonsteroidal anti-inflammatory drugs. History of tobacco exposure and use of medications. Patients were classified in asthma or no-asthma groups according to clinical symptoms and the pneumonologist’s evaluation. Also, patients were asked about the presence of the following nasal symptoms: nasal obstruction, nasal secretion, snoring, crusting, olfactory alterations, facial pain, and noisy breathing. Patients were also asked to grade the nasal obstruction with a Visual Analog Scale (VAS). Finally, all patients answered the Sino-Nasal Outcome Test-22 (SNOT-22)^19^ Nasal endoscopy was graded according to the Lund-Kennedy score (LK). Sinus CT scans were graded according to the Lund-Mackay score (LM).

### Pulmonary function tests

The device used for lung function tests was the Master Screen impulse oscillometry (Erich Jaeger, Germany®), located at the Clinics Hospital (UNICAMP). The system was calibrated with a three-liter syringe before each data collection session. Patients performed spirometry following the American Thoracic Society (ATS) guidelines.^20^ Additionally, patients underwent impulse oscillometry testing according to European Respiratory Society (ERS) guidelines.^17^^,^^21^ The impulse oscillometry assesses respiratory mechanics by applying pressure oscillations during tidal breathing, allowing measurement of airway resistance and reactance at various frequencies. Resistance (R) indicates opposition to airflow: R5 represents total resistance (central and peripheral airways), while R20 is more closely associated with central airways; the difference R5–R20 indicates peripheral resistance. Reactance (X) reflects the elasticity and inertia of the respiratory system: X5, measured at 5 Hz, becomes negative when compliance is reduced or air trapping occurs, indicating peripheral alterations. Fres (resonant frequency) is the frequency at which reactance equals zero (X = 0), with higher values suggesting increased lung stiffness or peripheral obstruction. AX (area of reactance) is the area under the reactance curve from 5 Hz to Fres, serving as a global index of peripheral respiratory load ‒ the larger the value, the more impaired the distal lung function.^21^

### Biomarkers

All patients had blood drawn for eosinophil and serum total IgE counts. Additionally, a Skin Prick test was performed evaluating the following allergens: *Dermatophagoides pteronyssinus, Dermatophagoides farina, Blomia tropicalis, cat* epitelium*, Dog* epiteliun*, Blatella germanica, Periplaneta americana,* and fungi. Also, all patients underwent polyp biopsy and pathological evaluation, differentiating between high- and low-eosinophil infiltrates. This biopsy was used for inflammatory phenotyping and sample characterization, and not as a primary variable for comparative statistical analyses. Patients were asked to withdraw systemic and local nasal steroids, including sprays combined with other medications, 30 days before the visit due to their possible interference with the biopsy results. Patients were classified into type 2 and non-type 2 according to the EPOS criteria (Tissue eosinophils ≥ 10 high power field, OR blood eosinophils ≥ 250 μ/L, OR total IgE ≥ 100 IU/mL).^3^

Blood eosinophilia was defined according to the 2020 EPOS criteria for type 2 inflammatory endotype classification, which were primarily developed for phenotypic characterization. Although the 2023 EPOS/EUFOREA update proposes a lower cut-off (≥150 cells/μL) primarily for identifying candidates for biologic therapy, a sensitivity analysis was performed using this updated threshold.^22^ With the ≥ 150 cells/μL criterion, five patients were reclassified from the non-type 2 to the type 2 group; however, the comparative functional analyses yielded similar results, with no statistically significant differences between groups.

### Statistical analysis

The sample size calculation was based on a comparison of FEV₁ (%) between patients with and without asthma. A clinically relevant difference of 15% was assumed, with an estimated standard deviation of 16.5% based on previously published data in patients with chronic rhinosinusitis.^23^ Considering a two-tailed α of 0.05 and 80% power, the required sample size was 21 patients per group (42 total), with asthma and no asthma groups, using the Mann-Whitney test as the nonparametric test. It was decided to increase the number to 52 patients, taking into account potential difficulties in performing pulmonary function tests.

Data were analyzed with SPSS 16.0 and STATA 12.0 software. Qualitative variables are presented in tables containing absolute and relative frequencies. The Shapiro-Wilk test was used to assess the normality of quantitative variables. For normally distributed variables, the Student's *t*-test was used to compare two independent groups; otherwise, the Mann-Whitney test was used. The Spearman Correlation Coefficient was used to assess the correlation between quantitative variables. Correlation figures were done using GraphPad Prism version 10.0.0 for MacOS, GraphPad Software, Boston, Massachusetts USA, www.graphpad.com. In all analyses developed, the significance level was set at 5%.

## Results

A total of 52 patients were evaluated, of whom 29 were male (55.8%). The main characteristics of the patients enrolled in the study are described in Table 1. The pulmonary function tests demonstrated generally preserved lung volumes and flows, with a mean FVC of 3.77 L (94.88% predicted) and a FEV1 of 2.84 L (88.05% predicted). The FEV1/FVC ratio averaged 73.62% and FEF25‒75% was 76.82%. Impulse oscillometry revealed resistance values with a mean resistance at 5 Hz (R5) of 122.72% and resistance at 20 Hz (R20) of 112.53% predicted, along with impedance of 128.7% and reactance of −0.15 kPa/L/s. Also, the area of reactance (AX), was increased with a mean value of 1.1 kPa/L. Supplementary material (Appendix A) shows the mean findings of the spirometry and impulse oscillometry.

**Table 1:** Demographic characteristics of patients included in the study.

Age (years)	54.7 ± 14.5**
Male	29 (55.8%)*
Asthma	24 (46.2%)*
Smokers	3 (5.8%)*
NSAID intolerance	8 (15.4%)*
Previous surgery	
None	27 (51.9%)*
1	16 (30.8%)*
>2	9 (17.3%)*
Comorbities	
Systemic arterial hypertension	24 (46.2%)*
Type 2 diabetics	6 (11.5%)*
Chronic renal disease	1 (1.9%)*
Psychiatry disease	5 (9.6%)*
Hypothyroidism	9 (17.3%)*
Type 2 Inflammation	42 (80.8%)*
Positive Prick test	19 (36.5%)*
Seric eosinophils	450 ± 350**
IgE	255.7 ± 71.2**
SNOT-22	48.35 ± 49.5**
Lund-Kennedy (LK)	7.4 ± 8**
Lund-Mackay (LM)	14.9 ± 15**
Visual Analogue Scale (VAS) – nasal obstruction	57.4 ± 55**

* Categorical data are presented as absolute (N) and relative (%) frequencies.

** Numerical data are presented as mean ± standard deviation.

**Fig. 1:**
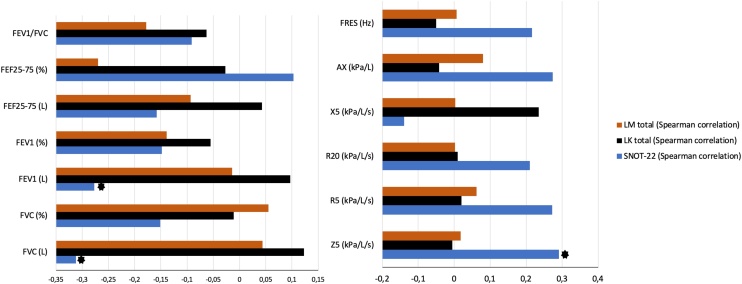
Correlation between upper airway severity and impulse oscillometry and spirometry parameters. (٭) Shows significant negative correlation between SNOT-22 and FEV1 (*p* = 0.047) and FVC (*p* = 0.024), and, and positive correlation between Z5 and SNOT-22 (*p* = 0.04). FVC, Forced Vital Capacity; FEV1, Forced Expiratory Volume in 1 s; FEF25%–75%, Forced Expiratory Flow at 25%–75% of FVC; PEF, Peak Expiratory Flow; FEV1/FVC, Ratio between FEV1 and FVC; SNOT, Sinunasal Outcome Test; LM, Lund-Mackay; LK, Lund Kennedy Z5, impedance at 5 Hz; R5, Resistance at 5 Hz; R20, Resistance at 20 Hz; X5, 5 Hz reactance; Fres, Resonant Frequency; Hz, Hertz; AX, Reactance Area; kPa/L/s, Unit of measurement in Kilopascal per liter per second; kPa/L, Kilopascal per Liter.

Fig. 1 illustrates the correlation between lung function tests and the severity of chronic rhinosinusitis, using the SNOT-22 score, LM score, and LK score. There was a negative correlation between SNOT-22 scores and FCV and Forced Expiratory Volume in 1 s (Forced Expiratory Volume [FEV]1) parameters. No correlation was found between spirometry parameters and LK and LM scores. Considering the impulse oscillometry correlations with these same parameters, a positive correlation was observed between SNOT-22 and Impedance at 5 Hz (Z5).

**Fig. 2:**
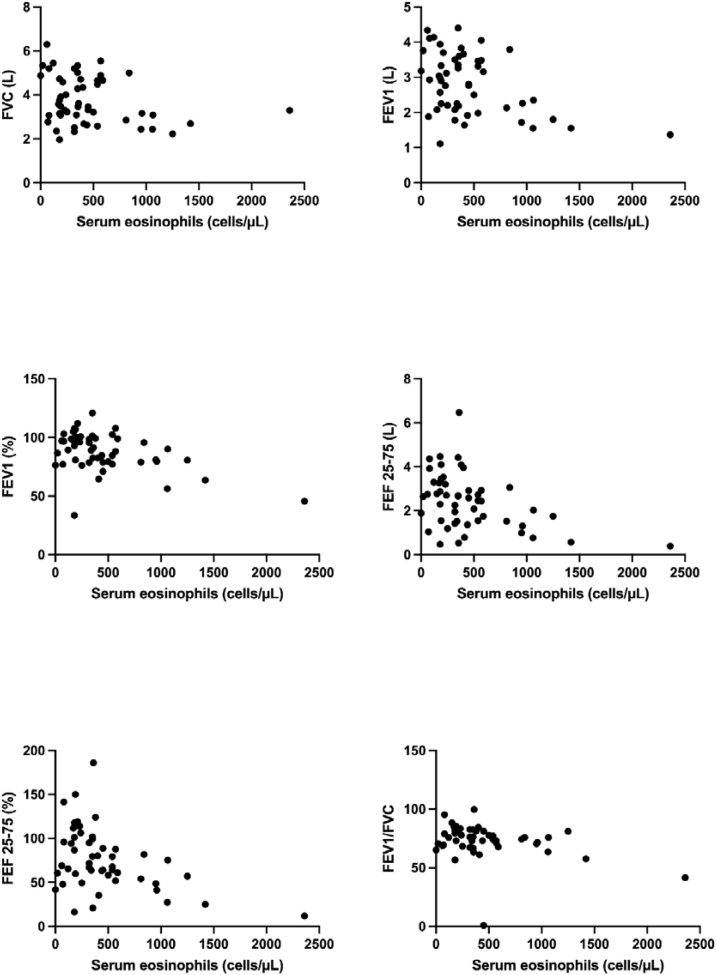
Correlation between Eosinophils and spirometric parameters: A negative correlation was observed between eosinophils and FEV1, FEF25–75%, and FEV1/FVC. FVC, Forced Vital Capacity; FEV1, Forced Expiratory Volume in 1 s; FEF25–75%, Forced Expiratory Flow at 25–75% of FVC; PEF, Peak Expiratory Flow; FEV1/FVC, ratio between FEV1 and FVC.

**Fig. 3:**
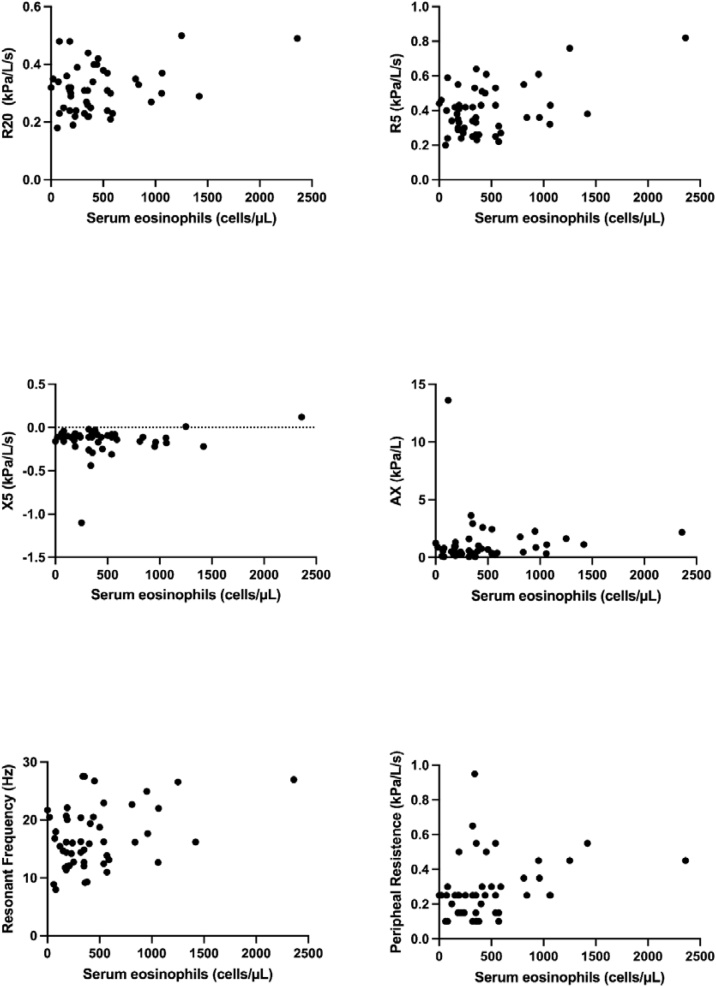
Correlation between Eosinophils and Impulse Oscillometry parameters: A positive correlation was observed between peripheral resistance and eosinophils. Serum eosinophils (cells/μL); R5, Total resistance; R20, Central resistance; X5, 5 Hz reactance; Fres, Resonant Frequency; Hz, Hertz; AX, Reactance Area; kPa/L/s, Unit of measurement in Kilopascal per liter per second; kPa/L, Kilopascal per liter.

Fig. 2 shows the correlation analysis between blood eosinophils and spirometry. As for the inflammatory profile of those patients, there was a negative correlation between total Eosinophils and FEV1 (*r* = −0.313; *p* = 0.024), FEV1% (*r* = −0.316; *p* = 0.023), Forced Expiratory Flow at 25–75% of FVC (FEF25–75%) (*r* = −0.38; *p* = 0.005), Forced Expiratory Flow at 25–75% of FVC (FEF25–75%)% (*r* = −0.37; *p* = 0.007) and FEV1/Forced Vital Capacity (FVC) (*r* = −0.287; *p* = 0.039). There was no correlation between Total IgE and spirometry parameters. There was no correlation between total IgE and impulse oscillometry parameters. However, there was a positive correlation between total Eosinophils and peripheral resistance (R5‒R20) parameters (*r* = 0.338; *p* = 0.018), as shown in Fig. 3.

There was no statistically significant difference in the distributions of impulse oscillometry and spirometry parameters between the groups with and without aeroallergen sensitization as determined by the Prick Test. As expected, there was a correlation between spirometry changes and patients with a clinical diagnosis of asthma, regardless of whether they had a positive or negative prick test. However, when patients were divided into type 2 and non-type 2 phenotype groups, there was no difference in spirometry or impulse oscillometry parameters (Table 2).

**Table 2:** Spirometry and impulse oscillometry parameters according to inflammatory phenotype.

Spirometry	Non-Type 2 (*n* = 10)		Type 2 (*n* = 42)		p-value
	Mean	SD	Mean	SD	
FVC (L)	4.04	1.14	3.70	1.06	0.347
FVC (%)	91.4	17.1	95.7	14.0	0.935
FEV1 (L)	3.14	0.92	2.76	0.85	0.174
FEV1 (%)	90.6	22.2	87.4	14.8	0.190
FEV25‒75% (L/s)	2.95	1.20	2.33	1.25	0.092
FEV25‒75%	91.6	41.2	73.3	32.9	0.137
FEV1/FVC	77.36	10.02	72.73	14.93	0.365
**Impulse oscillometry**					
Z5 Impedance (kPa/L/s)	0.37	0.11	0.44	0.16	0.178
Z5 Impedance (%)	122.1	32.8	130.3	41.4	0.671
R5 Resistance (kPa/L/s)	0.36	0.11	0.41	0.15	0.243
R5 Resistance (%)	118.7	33.6	123.6	39.7	0.816
R20 Resistance (kPa/L/s)	0.29	0.08	0.32	0.09	0.243
R20 Resistance (%)	111.1	28.9	112.9	28.6	0.846
X5 Reactance (kPa/L/s)	0.10	0.03	0.16	0.18	0.348
X5 Reactance (%)	145.2	593.6	305.6	1608.3	0.923
AX Reactance area (kPa/L)	1.82	4.16	0.93	0.89	0.628
Fres Resonant frequency (Hz)	15.48	4.17	17.34	5.48	0.396
Central Resistence (kPa/L/s)	0.17	0.10	0.22	0.09	0.189
Peripheral Resistence (kPa/L/s)	0.20	0.06	0.30	0.18	0.09

FVC, Forced Vital Capacity; FEV1, Forced Expiratory Volume in 1 second; FEF25–75%, Forced Expiratory Flow at 25–75% of FVC; PEF, Peak Expiratory Flow; FEV1/FVC, ratio between FEV1 and FVC. R5, Total resistance; R20, Central resistance; X5, 5 Hz reactance; Fres, Resonant frequency; Hz, Hertz; AX, Reactance Area; kPa/L/s, Unit of measurement in Kilopascal per liter per second; kPa/L, Kilopascal per liter.

When nasal obstruction was evaluated using the Visual Analogue Scale (VAS), it did not correlate with any of the lung function parameters, inflammatory biomarkers, or CRS severity markers.

Clinical characteristics, inflammatory scores, and pulmonary function parameters were compared between patients with (*n* = 24) and without a diagnosis of asthma (*n* = 28) (Table 3). No statistically significant differences were observed between the groups regarding sex (*p* = 0.18), age (*p* = 0.56), or body mass index (*p* = 0.22). Statistically significant differences were observed across several pulmonary function parameters. Asthmatic patients presented significantly lower values of FEV1 (2.51 ± 0.9 L vs. 3.11 ± 0.74 L; *p* = 0.011), and FEV1/FVC (*p* = 0.011). They also showed lower values for FEF25–75% (2.97 ± 1.22 L vs. 1.84 ± 0.99; *p* < 0.001), indicating impaired function peripheral airways.

**Table 3:** Comparison between asthmatic and non-asthmatic patients according to CRS gravity, inflammatory biomarkers, and Lung function parameters.

	Asthma (*n* = 24)	No Asthma (*n* = 28)	p-value
Male	11 (45.8%)	18 (64.3%)	0.182
Age (mean ± SD)	55.9 ± 13.8	53.6 ± 14.3	0.560
Type 2 phenotype	21 (87.5%)	21 (75%)	0.254
Previous Surgery	10 (41.7%)	15 (53.6%)	0.392
BMI (mean ± DP)	26.9 ± 4.3	28.4 ± 4	0.222
Positive Prick test	10 (41.6%)	9 (32.1%)	0.477
Lund-Mackay score	17.8 ± 4.8	12.3 ± 5.4	**<0.001**
Lund-Kennedy Score	7.7 ± 2.7	7.2 ± 2.5	0.573
SNOT-22	46.1 ± 24.6	50.2 ± 27.2	0.579
IgE	216.0 ± 301.0	289.7 ± 545.0	0.826
Eosinophils	576.0 ± 524.0	341.2 ± 264.0	0.072
FVC (L)	3.52 ± 1.11	3.98 ± 1.00	0.126
FEV1 (L)	2.51 ± 0.90	3.11 ± 0.74	**0.011**
FEV1/FVC	67.37 ± 16.90	78.97 ± 8.28	**0.002**
FEF25-75% (L/s)	1.84 ± 0.99	2.97 ± 1.22	**<0.001**
Z5 (kPa/L/s)	0.44 ± 0.15	0.41 ± 0.14	0.395
R5 (kPa/L/s)	0.41 ± 0.14	0.39 ± 0.14	0.526
R20 (kPa/L/s)	0.31 ± 0.07	0.31 ± 0.09	0.919
X5 (kPa/L/s)	−0.14 ± 0.11	−0.15 ± 0.19	0.179
Fres Resonant frequency (Hz)	17.62 ± 5.61	16.4 ± 5.00	0.434
AX (kPa/L)	1.02 ± 0.94	1.16 ± 2.53	0.363
Central resistence (kPa/L/s)	0.23 ± 0.09	0.2 ± 0.09	0.323
Peripheral resistence (kPa/L/s)	0.32 ± 0.2	0.25 ± 0.12	0.215

FVC, Forced Vital Capacity; FEV1, Forced Expiratory Volume in 1 s; FEF25–75%, Forced Expiratory Flow at 25–75% of FVC; FEV1/FVC, ratio between FEV1 and FVC.

R5, Total resistance; R20, Central resistance; X5, 5 Hz reactance; Z5, Respiratory impedance at 5 Hz; Fres, Resonant frequency; Hz, Hertz; AX, Reactance Area; kPa/L/s, Unit of measurement in Kilopascal per liter per second; kPa/L, Kilopascal per liter.

Additionally, patients with asthma had higher Lund-Mackay scores (17.83 ± 4.77 vs. 12.29 ± 5.43; *p* ≤ 0.01), suggesting more severe sinonasal inflammation. However, there was no difference in impulse oscillometry parameters in asthma and non-asthmatic patients.

## Discussion

This was the first study to investigate pulmonary function alterations in patients with chronic rhinosinusitis using impulse oscillometry in combination with spirometry. There is a lack of standardization of impulse oscillometry values in adults; however, patients with CRS in our study had a higher Resonant Frequency (above 12), which has been reported in both restrictive and obstructive lung diseases.^16^^,^^17^^,^^21^ Evidence suggests that small airway dysfunction may define a distinct asthma phenotype, present across all disease stages, and linked to poor control. Alternatively, it could act as an early marker of asthma rather than a separate phenotype.^24^, ^25^, ^26^

Li et al. evaluated cut-off values of impulse oscillometry that identified patients with small airway diseases and normal spirometry, and found that values higher than 0.30 kPa/L/s for R5, 0.015 kPa/L/s for R5–R20, 0.30 kPa/L for AX, and 11.23 Hz for Fres were compatible with this asthma phenotype.^25^ Considering these values, participants of our study without a diagnosis of asthma would present alterations associated with small airway disease. Lipworth et al. determined more conservative cut-off values to identify small airway disease, but even with these cut-offs, our results still show small airway disease in patients without asthma.^24^^,^^26^

When patients with and without asthma were compared in this study, spirometry values differed; however, impulse oscillometry parameters did not differ significantly. Impulse oscillometry has been shown to be more sensitive to obstruction than spirometry, and the similarity in results between patients with and without asthma may indicate underlying asymptomatic lung obstruction.^15^^,^^16^^,^^21^ Kim et al. demonstrated that a cut-off point of 0.51 in the impulse oscillometry parameter AX (area of reactance) was sensitive for diagnosing asthma in patients with preserved lung function, and non-asthmatic patients had a mean AX of 1.16 in our study.^15^ Although the resistance in impulse oscillometry was lower than 150%, a previous parameter used in literature, both patients with and without asthma had similar small airway resistance in impulse oscillometry.^16^,^27^ In our population of patients with chronic rhinosinusitis, demographic characteristics of the patients, and LK, LM, and SNOT-22 were very similar to a recent large study. Among patients with nasal polyps, there is a similar rate of inflammatory type 2 phenotype, of more than 80%.^28^

There was a negative correlation between FVC and FEV1 and SNOT-22, what can be interpreted by higher the severity of symptons, worst the lung function parameters, and there was a correlation with Z5 impedance at 5 Hz, meaning that higher the severity of symptoms there is greater overall airway obstruction or increased airway wall stiffnes, as the Z5 impedance includes both airway resistance and reactance, at a low frequency (5 Hz).

Previous research demonstrated that patients with chronic rhinosinusitis have an impaired small airway function, especially a FEF25–75% decrease.^12^^,^^23^ Also, a systematic review that evaluated the impact of functional sinus surgery on lung function demonstrated an improvement in FEF25–75% after this intervention in patients with chronic rhinosinusitis.^11^ Zhao et al. evaluated patients with chronic cough and chronic rhinosinusitis and found significant decreases in FEV1 and FEV/FVC compared with controls. Prior studies indicate that spirometry measurements are less sensitive for evaluating small airway disease; therefore, impulse oscillometry can be a useful tool to complement airway investigation.^21^ Resistance at 5 Hz–20 Hz (R5‒R20) parameters in impulse oscillometry indicate small airway diseases, and, in our study, there was a positive correlation between peripheral lung resistance and eosinophil count.

Nasal obstruction, as measured by the VAS, did not correlate with poorer lung function. A previous study by Kariya et al. also demonstrated no correlation between nasal obstruction, as measured by rhinomanometry, and lung alterations on spirometry.^23^ Also, allergen sensitization did not correlate with the worst lung function, as assessed by spirometry or impulse oscillometry, indicating that allergy was not the predominant factor associated with lower and upper airway diseases in patients with chronic rhinosinusitis. Although asthma and rhinitis have a well-established relationship, the chronic rhinosinusitis phenotype, characterized by a high IL-5 response, stimulates eosinophilic responses but not necessarily IgE production.^29^^,^^30^

A previous study showed that IgE levels and CT score did not significantly correlate with spirometry parameters.^14^ IgE did not correlate with lung function in previous studies evaluating lung function in patients with chronic rhinosinusitis, even though high IgE levels are considered an important biomarker for classifying both asthma and chronic sinusitis phenotypes.^4^^,^^9^^,^^12^^,^^14^^,^^23^^,^^31^ There is a current debate in the literature about whether IgE levels are a good biomarker in chronic rhinosinusitis, as they can be elevated in monoclonal form in allergic rhinitis despite the presence of chronic rhinosinusitis.^32^

Moreover, previous reports found no correlation between eosinophil blood counts and alterations in lung function in patients with chronic rhinosinusitis.^14^^,^^23^^,^^33^ The literature presents conflicting views on whether eosinophil blood counts serve as a biomarker of asthma severity, as various studies yield mixed results, possibly due to phenotypic variation and changes in blood eosinophil counts associated with systemic steroid use.^34^, ^35^, ^36^ Du et al. demonstrated that lower airway dysfunction is associated with upper airway disease and the level of type-2 inflammation, as indicated by blood and nasal IgE levels and eosinophil counts.^28^ Our study revealed no significant differences in spirometry and impulse oscillometry parameters between patients with type 2 and non-type 2 chronic sinusitis phenotypes. The small number of patients without a type 2 phenotype may justify the lack of difference between groups. Nevertheless, this might be attributed to differing inflammatory endotypes within the type 2 phenotype in chronic rhinosinusitis patients.^2^^,^^9^ Also, evidence suggests that the highest levels of peripheral blood eosinophils and/or eosinophilic infiltration in nasal polyp tissue occur in individuals with both upper and lower airway diseases, in contrast to those with isolated severe eosinophilic asthma or chronic rhinosinusitis with nasal polyps.^9^^,^^37^

Furthermore, it is essential to highlight some limitations of this study; this was a single-center study, with most of the analyzed patients having type 2 phenotype, due to a predominant characteristic in our population, making it difficult to extrapolate results to the entire population and generalize conclusions. This may be a sign of a need for a higher cut-off point to distinguish eosinophilic CRS, or a higher type 2 endotype subclassification.^13^^,^^28^^,^^38^ Also, our study did not distinguish endotypes that are probably more or less associated with lung function alterations. A bigger sample size would increase the power of this study and better differentiate phenotypes associated with lower airway dysfunction. Also, the cross-sectional methodology can’t infer causality, and a small sample size can decrease the study's power when analyzing multiple variables. However, as an exploratory study, this article is a vital hypothesis generator within the unified airway theory.

Bousquet et al. recently classified single-airway diseases (isolated rhinitis, sinusitis, asthma, and COPD), linked to non-T2 endotypes, and one-airway-one disease when upper-lower airway diseases are combined, typically driven by T2 inflammation.^39^ The specific roles of eosinophils and IL-5 in the pathophysiology of asthma and Chronic Rhinosinusitis with Nasal Polyps (CRSwNP) are distinct. Although the upper and lower airways share the same inflammatory biomarkers, these biomarkers do not necessarily correlate with disease severity or diagnosis. That may be reflected in the efficacy observed with eosinophil- and IL-5/targeted biologics in patients with asthma compared to those with CRSwNP.^9^

## Conclusion

Impulse oscillometry parameters may be compatible with small airway disease in patients with chronic rhinosinusitis despite the diagnosis of asthma. Moreover, this study demonstrated a negative correlation between spirometry parameters and eosinophil blood count. There was also a negative correlation between the severity of CRS symptoms and FEV1 and FVC, and between LM score and impulse oscillometry parameters. IgE did not correlate with lung function alterations despite its use in phenotyping both asthma and chronic rhinosinusitis.

## ORCID IDs

Mariana Dalbo Contrera Toro: 0000-0002-5294-3698

Carla Cristina Souza Gomez: 0000-0002-3878-7772

Paloma Lopes Francisco Parazzi: 0000-0002-7072-603X

Maria Ângela Goncalves Oliveira Ribeiro: 0000-0002-9418-1919

André Moreno Morcillo: 0000-0002-2088-972X

José Dirceu Ribeiro: 0000-0002-3387-5642

Eulália Sakano: 0000-0002-5963-912X

## CRediT authorship contribution statement

MDCT wrote the manuscript, performed the otorhinolaryngology tests, analyzed and interpreted the data; CCSG and PLFP performed the pulmonary function tests; MAGOR performed the pulmonary function tests and provided critical feedback and revisions; AMM did the statistical analyses; JDR and ES conceived and designed the study, and contributed to the interpretation of the results along with the critical review of the article.

## Data availability statement

The authors declare that all data are available in repository.

## Declaration of competing interest

There was no conflict of interest regarding this article and the authors who contributed to it.
