# Supplementary material for: Chronic rhinosinusitis association with small airway disease: Inflammatory biomarkers and lung function using impulse oscillometry
Source: Braz J Otorhinolaryngol. 2026 Jun 16;92(5):101842. doi: 10.1016/j.bjorl.2026.101842 (PMC13292784; doi:10.1016/j.bjorl.2026.101842)
Supplement: Supplementary file 1 [file mmc1.docx]

**BJORL-D-25-00335_Supplementary Material**

**Supplementary Material** Spirometry and IOS parameters distribution.

| **Parameter** | **Mean** | **95% CI Lower** | **95% CI Upper** | **Median** | **Standard Deviation** | **Min** | **Max** | **Range** | **Interquartile Range** |
| --- | --- | --- | --- | --- | --- | --- | --- | --- | --- |
| FVC (L) | 3.77 | 3.47 | 4.07 | 3.47 | 1.07 | 1.96 | 6.3 | 4.34 | 1.79 |
| FVC (%) | 94.88 | 90.82 | 98.94 | 95.7 | 14.58 | 46.0 | 131.0 | 85.0 | 15.43 |
| FEV1 (L) | 2.84 | 2.6 | 3.08 | 2.92 | 0.87 | 1.11 | 4.4 | 3.29 | 1.48 |
| FEV1 (%) | 88.05 | 83.52 | 92.59 | 89.65 | 16.29 | 33.5 | 120.8 | 87.3 | 20.1 |
| FEF25-75 (L) | 2.45 | 2.1 | 2.8 | 2.52 | 1.25 | 0.39 | 6.47 | 6.08 | 1.73 |
| FEF25-75 (%) | 76.82 | 67.08 | 86.55 | 70.45 | 34.97 | 11.8 | 186.1 | 174.3 | 43.5 |
| FEV1/FVC (%) | 73.62 | 69.68 | 77.56 | 75.8 | 14.15 | 0.83 | 99.64 | 98.81 | 12.41 |
| Z5 Impedance at 5 Hz (%) | 128.7 | 117.43 | 139.97 | 121.6 | 39.65 | 61.4 | 238.7 | 177.3 | 59.35 |
| R5 Resistance at 5 Hz (Raw) | 0.4 | 0.36 | 0.44 | 0.38 | 0.14 | 0.2 | 0.82 | 0.62 | 0.19 |
| R5 Resistance at 5 Hz (%) | 122.72 | 111.69 | 133.75 | 115.7 | 38.4 | 62.7 | 236.4 | 173.7 | 60.6 |
| R20 Reactance at 20 Hz (Raw) | 0.31 | 0.29 | 0.34 | 0.31 | 0.08 | 0.18 | 0.5 | 0.32 | 0.13 |
| R20 Resistance at 20 Hz (%) | 112.53 | 104.46 | 120.59 | 108.05 | 28.39 | 69.6 | 174.1 | 104.5 | 42.05 |
| X5 Reactance at 5 Hz (Raw) | -0.15 | -0.19 | -0.1 | -0.11 | 0.16 | -1.1 | 0.12 | 1.22 | 0.08 |
| AX reactance area | 1.1 | 0.54 | 1.67 | 0.54 | 1.98 | 0.06 | 13.62 | 13.56 | 0.85 |
| Resonant Frequency (Hz) | 16.97 | 15.47 | 18.46 | 16.17 | 5.25 | 7.99 | 27.53 | 19.54 | 7.87 |

FVC, Forced Vital Capacity; FEV1, Forced Expiratory Volume in 1 second; FEF25–75%, Forced Expiratory Flow at 25–75% of FVC; PEF, Peak Expiratory Flow; FEV1/FVC, ratio between FEV1 and FVC.
